# Unexpected chronic lymphocytic leukemia B cell activation by bisphosphonates

**DOI:** 10.1007/s00262-023-03588-z

**Published:** 2024-01-27

**Authors:** Andrea N. Mazzarello, Elena Gugiatti, Vanessa Cossu, Nadia Bertola, Davide Bagnara, Sonia Carta, Silvia Ravera, Chiara Salvetti, Adalberto Ibatici, Fabio Ghiotto, Monica Colombo, Giovanna Cutrona, Cecilia Marini, Gianmario Sambuceti, Franco Fais, Silvia Bruno

**Affiliations:** 1 Department of Experimental Medicine (DIMES), University of Genoa, Via De Toni 14, 16132 Genoa, Italy; 2 Molecular Pathology Unit, IRCCS Ospedale Policlinico San Martino, Genoa, Italy; 3 Nuclear Medicine Unit, IRCCS Ospedale Policlinico San Martino, Genoa, Italy; 4 Clinic of Hematology, IRCCS Ospedale Policlinico San Martino, Genoa, Italy; 5 Division of Hematology and Bone Marrow Transplant, IRCCS Ospedale Policlinico San Martino, Genoa, Italy; 6 Institute of Molecular Bioimaging and Physiology (IBFM), National Research Council (CNR), Milan, Italy; 7 Department of Health Sciences (DISSAL), University of Genoa, Genoa, Italy

**Keywords:** Chronic lymphocytic leukemia (CLL), Bisphosphonates, CLL cell activation, CLL cell treatment

## Abstract

Chronic lymphocytic leukemia (CLL) is a disease of the elderly, often presenting comorbidities like osteoporosis and requiring, in a relevant proportion of cases, treatment with bisphosphonates (BPs). This class of drugs was shown in preclinical investigations to also possess anticancer properties*.* We started an in vitro study of the effects of BPs on CLL B cells activated by microenvironment-mimicking stimuli and observed that, depending on drug concentration, hormetic effects were induced on the leukemic cells. Higher doses induced cytotoxicity whereas at lower concentrations, more likely occurring in vivo, the drugs generated a protective effect from spontaneous and chemotherapy-induced apoptosis, and augmented CLL B cell activation/proliferation. This CLL-activation effect promoted by the BPs was associated with markers of poor CLL prognosis and required the presence of bystander stromal cells. Functional experiments suggested that this phenomenon involves the release of soluble factors and is increased by cellular contact between stroma and CLL B cells. Since CLL patients often present comorbidities such as osteoporosis and considering the diverse outcomes in both CLL disease progression and CLL response to treatment among patients, illustrating this phenomenon holds potential significance in driving additional investigations.

**Supplementary Information:**

The online version contains supplementary material available at 10.1007/s00262-023-03588-z.

## Introduction

Chronic Lymphocytic Leukemia (CLL) is the most common B-cell malignancy among European descent and is characterized by the monoclonal expansion of CD5 + B cells. The clinical course of CLL patients is highly heterogeneous with some cases surviving for decades without therapeutic intervention while others require treatment shortly after diagnosis. In the last decade, advancements in the therapeutic armamentarium have highly improved the clinical management of this disease. Despite that, CLL remains incurable [1]. Importantly, CLL is infrequent among individuals ≤ 40 years old, as the average diagnosis age ranges from 65 to 70 years [2, 3]. Consequently, CLL patients frequently exhibit concurrent health issues, which are linked to worse prognoses and necessitate simultaneous treatment for these diverse conditions [4, 5]. Osteoporosis, among the conceivable concurrent conditions, may also be prompted or exacerbated by the leukemic cells [6, 7]. As of now, there are not comprehensive investigations regarding the overall number of CLL patients undergoing treatment for osteoporosis. Nevertheless, it could range between 12.5 and 31% of the total CLL patients, as respectively found in two independent studies [8, 9].

Bisphosphonates (BPs), a class of drugs commonly prescribed for the treatment of osteoporosis, are stable analogues of pyrophosphates. BPs are divided into two classes, the N-containing and non-N-containing bisphosphonates, targeting osteoclast cells with different mechanisms of action [10]. Specifically, the N-containing bisphosphonates, like Zoledronate (ZOL) and Pamidronate (PAM), induce apoptosis in osteoclasts by inhibiting the mevalonate pathway, preventing protein prenylation, thus disrupting osteoclast cytoskeleton remodeling and inhibiting the resorptive function of osteoclast [10]. The non-N-containing bisphosphonates, like Clodronate (CLO) and Etidronate (HEDP), are metabolically converted to non-hydrolyzable toxic ATP analogues, which impair a variety of metabolic processes and thereby induce osteoclast apoptosis [10, 11].

However, BPs effects are likely not circumscribed to targeting bone loss, as they may affect tumor cells as well. Recent preclinical studies in various types of cancer indicate that BPs exhibit an antitumoral activity exerted, either directly, through the inhibition of the mevalonate pathway in cancer cells [12], or indirectly, through different mechanisms, including antiangiogenic activity [13], depletion of pro-tumoral macrophages from the microenvironment [14] and their polarization shift to antitumor M1 phenotype [15] and, for ZOL, increased cancer immune surveillance through activation of γδ T cells [13, 16]. In line with these studies, cytotoxic effects of both N-containing (ZOL) and non-N-containing (PAM) BPs were also documented in vitro on leukemic cells obtained ex vivo from patients with CLL [17].

Accordingly, a significant number of CLL patients presenting osteoporosis is likely undergoing treatment with BPs that could affect the leukemic clone as well. Of note, most of the previous studies, showing cytotoxicity in cancer cells, were conducted using BPs concentrations that are unlikely observable outside of calcified issues, such as lymph nodes and other secondary lymphoid tissues [18].

In this view, since the microenvironment plays a key role in the pathogenesis and clonal expansion of CLL B cells, by supporting the activation and proliferation of intraclonal subpopulation(s) of the leukemic clone [19–21], identifying the effects of BPs in these proliferative CLL B subpopulations might improve our therapeutic abilities. For these reasons, we investigated the role of BPs in CLL B cells activated through microenvironment stimuli.

## Materials and methods

### Cells and cell cultures

CLL cells were obtained from the peripheral blood of CLL patients, after informed consent according to the Declaration of Helsinki. Mononuclear cells separated by Ficoll density gradient centrifugation were assayed by flow cytometry (FACSCalibur, BD Biosciences, San Diego, CA) for standard diagnostic immunophenotyping. For all experiments, naïve PBMCs from CLL patients with at least 85% CD5^+^CD19^+^ CLL B cells were used. CLL B cells purification was carried out by immunomagnetic negative selection using the EasySep Direct Human B-CLL Cell Isolation Kit (Stemcell Technologies, Catalog # 19664), as per manufacturer instructions. A comprehensive list of the CLL cohort with clinical and molecular features is reported in the Supplementary Table S1.

CLL cells were cultured in RPMI culture medium with 10% FBS at high cell density, 2–4 × 10^6^/ml. Activation of CLL cells through CD40 was achieved by co-culturing CLL cells in the presence of a stable CD40L-expressing NIH-3T3 murine fibroblast cell line produced in our laboratory, at a cell number ratio 1:100 (fibroblasts:CLL cells), with the addition of IL-4 (10 ng/ml). Activation by CpG/ODN2006 (hTLR9 ligand) was performed by the addition of these unmethylated CpG dinucleotides at the concentration of 2 μg/ml with the addition of IL-15 (10 ng/ml). The following BPs were from Merck (KGaA, Darmstadt, Germany): Clodronate (dichloromethylene bisphosphonate), Zoledronate (1-hydroxy-2-[(1H-imidazole-1-yl) ethylidene] 1-bisphosphonate), Pamidronate (APD) (3-amino-1-hydroxy-propylidene-1,1-bisphosphonate), Risedronate (1-Hydroxy-2-(3-pyridinyl)ethylidene]bis[phosphonic acid). Etidronate (HEDP, Hydroxyethylidene diphosphonic acid) was produced and kindly provided by Italmatch Chemicals S.p.A. (https://www.italmatch.com). Drugs were added to stimulated CLL cells simultaneously to the activation stimuli.

Inhibition of the RANK-RANKL loop was achieved by the addition of the human monoclonal anti-RANKL antibody Denosumab (Xgeva, Amgen) to stimulated CLL cultures at a concentration of 730 nM. The 2′,3′-dialdehyde derivative of ATP, namely oxidized ATP (oATP) (Sigma Aldrich), was used as irreversible antagonist of the purinergic receptor P2X7R at the concentration of 100 μM. Positive controls were provided by treatment with ATP (Sigma-Aldrich) at 50 μM. The selective antagonist for the adenosine receptor P1A2A, SCH-58261 (Sigma Aldrich), was used at 2 μM. Positive controls were provided by treatment with 2-chloroadenosine (Sigma-Aldrich) at 50 μM.

### Flow-cytometric assays for apoptosis and proliferation.

Multiparameter flow-cytometric analysis of cellular, molecular and metabolic features were previously described: cellular viability by propidium iodide (PI) exclusion assays in Ponassi et al. [22], cell cycle-phase distribution by DNA content and expression of KI-67 in Bruno et al. [23], CFSE fluorescence dilution in Marini et al. [24].

### Flow cytometric evaluation of surface molecule expression

Analysis of surface protein levels was performed by immunofluorescence and flow cytometry on a FACS Calibur (Beckton Dickinson, San Josè, CA, USA). In selected experiments, the FACSCanto (BD Biosciences) flow cytometer was used.

Expression of adhesion/homing proteins was carried out by means of mouse anti-human anti-CD49d (integrin alpha4), anti-CD58 (LFA-3) and anti-CD69 monoclonal antibodies, all from BD Pharmingen (San Diego, CA). Secondary antibodies, from Molecular Probes (InVitrogen, Eugene, OR), were goat anti-mouse immunoglobulins conjugated to Alexa fluorochromes.

Expression of cell surface RANK and RANKL on CLL cells was assessed by flow cytometry analysis using RANK-PE (R&D System, clone#80704) and RANKL-APC (Biolegend, clone MIH24) monoclonal antibodies. It is important to highlight that the analysis of protein expression by flow cytometry was restricted to viable leukemic cells, namely cells falling within the ‘live’ gate on flow cytometric FSC-SSC plots, i.e. the FSC^high^ SSC^low^’ gate (thus gating out apoptotic and dead cells). This compartment contains CLL cells that have an intact plasma membrane and do not express activated caspase 3 [25]. CLL cells outside the gate are mostly late apoptotic and dead cells.

### Evaluation of ATP release in tissue culture medium

The extracellular ATP concentration in the tissue culture medium was determined after 5 days of CLO treatment of stimulated CLL samples with the ATP Bioluminescence Assay Kit HS II (Roche). Monocytes from healthy donors, obtained from the flask bottom-adherent fraction of peripheral blood mononuclear cells, were activated with 100 ng/ml LPS (from *Escherichia coli* 0111:B4; Sigma-Aldrich) for 18 h [26] and used as positive control.

### Statistics

Nonparametric statistics was applied, using the GraphPad Prism version 8 software (GraphPad Software Inc., La Jolla, CA). For each type of experiment and analysis the details are provided in Figure Legends. *p ≤ 0.05; **p ≤ 0.01; ***p ≤ 0.001; ***p ≤ 0.001; ****p ≤ 0.0001.

## Results

### BPs display in vitro dose-dependent pro-survival effects in CLL B cells

**Fig. 1:**
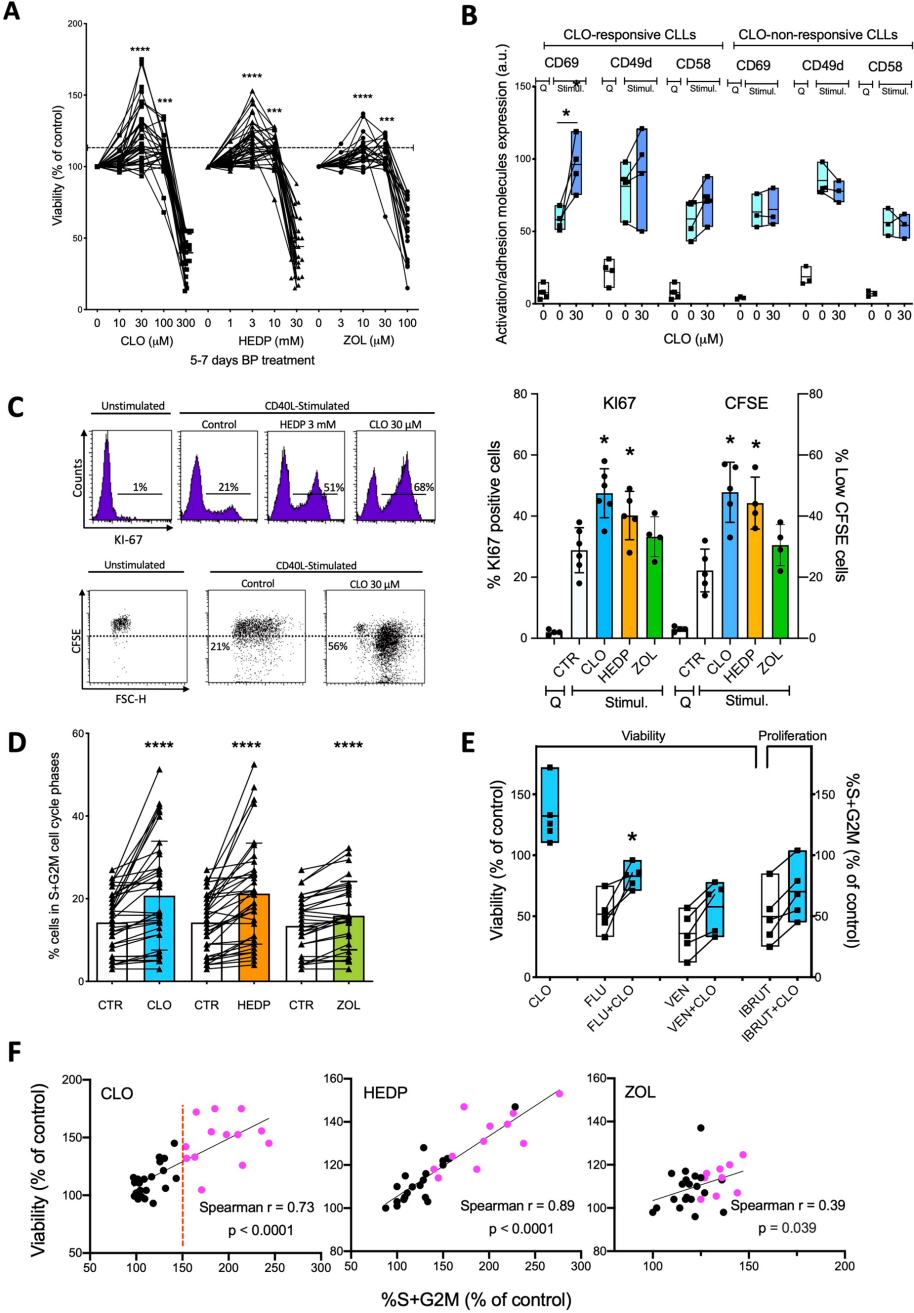
*BPs potentiate cell survival, activation, proliferation, and resistance to therapy of stimulated CLL cells.* **A** Cellular viability of n = 47 samples from n = 21 CLL patients (see Supplementary Table 1) cultured in presence of microenvironment-mimicking stimuli and BPs for 5–7 days. Viability of each BP-treated sample was assessed by Propidium Iodide (PI) exclusion assays and expressed as % of matched BP-untreated control. Dotted line indicates the value of 110%, defined as the operational threshold for the determination of ‘BP-mediated pro-survival effect’. **B** Plasma membrane levels of CD69, CD49d and CD58 of CLL samples selected among those that displayed ‘pro-survival’ response to BPs (n = 4, CLO-responsive pts # 5,8,11,12, left) and among those that did not (n = 3, CLO-unresponsive pts # 3,4,7, right). Samples were either unstimulated (Q) or stimulated with CD40L + IL-4 (Stimul.) and treated with 30 μM CLO for 5 days. **C** Effect of CLO, HEDP, and ZOL on CLL proliferation. Samples were selected among those displaying ‘pro-survival’ response to BPs and were either unstimulated (Q) or stimulated with CD40L + IL-4 (Stimul.) and treated with CLO (30 μM), HEDP (3 mM), or ZOL (10 μM) for 6 days. KI67 and CFSE fluorescence were analyzed by flow cytometry in n = 6 (pts # 5, 8, 11, 12, 14, 21), n = 5 (pts # 5, 8, 11, 12, 14), and n = 4 (pts # 8, 11, 12, 14) treated samples, respectively. Left side: selected flow cytometric dot plots; right side: cumulative data expressed as % of KI67^+^ cells (left Y axis) and % of cells with CFSE fluorescence below the ‘Quiescence’ threshold (right Y axis) are displayed as bars (mean ± SD). **D** Effect of BPs on cell proliferation as assessed by the % of cells in S + G2M cell cycle phases. N = 38 samples from n = 20 CLL patients were cultured with CD40L + IL-4 or CpG + IL-15 in the absence or presence of BPs (30 μM CLO, 3 mM HEDP or 10 μM ZOL). The percentage of cells in S + G2M was evaluated on flow cytometric DNA content histograms after 6 days of culture. **E** Left: cellular viability of n = 5 CLL samples selected among those displaying ‘pro-survival’ response to BPs (pt # 5, 8, 11, 12, 14). Samples were stimulated and cultured with Fludarabine (2.5 μM), Venetoclax (10 nM) in the absence or presence of CLO (30 μM), for 4 days. Viability of treated samples is expressed as % of untreated controls. Right: % S + G2M cells of 4 ‘CLO responder’ CLL samples stimulated and cultured with Ibrutinib (5 μM) for 4 days in the absence or presence of CLO. Proliferation of treated samples is expressed as % of untreated controls. Groups are shown as bars with data mean and range (min to max) and Wilcoxon matched-pairs test used to evaluate the significance of their difference. **F** Dot plots displaying the relationship between viability protection and proliferation potentiation exerted by each BPs (30 μM CLO, 3 mM HEDP or 10 μM ZOL) on stimulated CLL samples. Viability is expressed as % viable cells in BP-treated sample divided by % viable cells in control untreated sample (i.e., % of control). Proliferation is expressed as % S + G2M cells in BP-treated sample divided by % S + G2M cells in control untreated sample (i.e., % of control). Correlation analysis was performed using the non parametric Spearman correlation test. Dots in pink color represent high CLO-responder samples that were operationally and arbitrarily chosen on the CLO graph (left) as samples in which proliferation was increased by CLO by 50%. These same samples were then highlighted by pink color in the other two BP graphs (HEDP, middle, and ZOL, right)

At first, we evaluated the BPs direct cytotoxicity on leukemic B cells obtained *ex-vivo* from peripheral blood of CLL patients, either resting (as they are derived from the periphery) or stimulated in vitro by classical microenvironment-mimicking stimuli (CD40L + IL4, activating the CD40 pathway, or CpG + IL15, engaging the Toll-Like Receptor 9) [27]. After 2–3 days, a dose-dependent inhibitory effect on cell viability was observed for drug concentrations above 100 μM, 10 mM, and 30 μM for CLO, HEDP and ZOL, respectively (Supplementary Fig. S1). Following the cultures during the subsequent days, we noticed a different effect in the stimulated samples at BPs concentrations lower than the above-mentioned cytotoxic doses. Several of these samples were still viable after 10–20 days of treatment with BPs, whereas the untreated/stimulated and untreated/unstimulated samples did not contain any viable cell anymore. Thus, to address more in detail this observation, we activated CLL samples as above and followed them at longer time-points (5–7 days) after treatment with BPs. Instead of being cytotoxic, in a specific range of drug concentrations, namely around 30 μM for CLO, 3 mM for HEDP, and 10 μM for ZOL, the three BPs protected CLL B cells from spontaneous apoptosis (Fig. 1A). At higher concentrations the three BPs became cytotoxic.

We operationally chose a threshold above which a ‘BP-mediated pro-survival effect’ was defined, namely a level of viability of a BP-treated sample that exceeded by more than 10% the viability of the untreated control (i.e., > 110% of control). Accordingly, the ‘pro-survival’ phenomenon occurred in 27/47 (57.4%) CLO-treated, 22/46 (47.8%) HEDP-treated, and 8/30 (26.7%) ZOL-treated CLL B cell cultures activated by microenvironmental stimuli (Fig. 1A). Hence, these data support the contention that BPs at certain concentrations might enhance, in a significant number of CLL patients in vitro, a pro-survival response of the CLL B cells interacting with the microenvironment through various activation mechanisms.

### Pro-survival doses of BPs enhance activation and proliferation of BP-responding CLL B cells

Activation and adhesion receptors CD69, CD49d, and CD58, known for protecting cells from apoptosis and for associating with disease progression [28–30], were measured upon stimulation in four CLL samples previously identified as CLO-responding, and in three CLL samples that did not display any CLO-mediated protective effect (non-responsive). In the presence of CLO, increased membrane levels of CD69 were observed for 4/4 responding CLL samples (Fig. 1B). Similarly, CLO increased CD49d and CD58 membrane levels in 3/4 of the assayed CLL samples (Fig. 1B). Conversely, these BP effects were not observed in the non-responding subgroup (Fig. 1B). Hence, in the CLL BP-responders, the BP-induced protection from apoptosis appears to be linked with enhanced cellular activation.

The enhanced CLL survival/activation in presence of low BP concentrations was accompanied by increased entry in the cell cycle and subsequent proliferation, as confirmed by higher percentage of KI-67^+^ CLL B cells in CLO- and HEDP-treated cultures, and a trending increase in ZOL-treated cultures (Fig. 1C). Likewise, CFSE fluorescence decrement was higher in CLO-treated CLL samples compared to controls (Fig. 1C).

Analysis of the percentage of cells in S + G2M phases of the cell cycle, indicated that the ‘pro-proliferation’ effect was observed for all three BPs (Fig. 1D). Indeed, in more than half CLL CLO-treated samples (21/38, 55.2%), as well as in 24/37 (64.9%) HEDP-treated and in 13/25 (52%) ZOL-treated cultures, the proliferation levels exceeded those of the respective controls (Fig. 1D).

Since low concentrations of BPs enhance activated B cells survival and proliferation without displaying cytotoxicity, we questioned whether high BPs concentrations would produce similar effects in the CLL B cell fraction that did not undergo apoptosis yet. Thus, we measured cellular activation (CD69), cell cycle profile, and CFSE decay in the live fraction of activated CLL exposed to cytotoxic levels of CLO (300 μM), HEDP (30 mM), and ZOL (100 μM). While increased apoptosis was confirmed, no changes were observed, compared to the control, in the activation/proliferation patterns of the CLL cells that were still alive at the time of the analysis (Supplementary Fig. S2). Thus, high BPs concentrations were cytotoxic but did not display cytostatic features, nor enhancement of the proliferation in the remaining live CLL fraction (Supplementary Fig. S2), supporting the specificity of the BP-induced CLL activation enhancement only at low concentrations.

Because of the broad clinical use of BPs, we questioned whether the effectiveness of CLL therapy might be influenced. Thus, we tested the influence of CLO on CLL B cell response to i) the purine analogue Fludarabine; ii) the inhibitor of the anti-apoptotic B-cell lymphoma-2 (Bcl-2) protein, Venetoclax; iii) the inhibitor of the Bruton Tyrosine Kinase, Ibrutinib [31, 32].

Fludarabine-induced apoptosis was measured in samples displaying pro-survival enhancements in presence of BPs (BP-responsive samples). Fludarabine alone drastically decreased viability, as expected. In contrast, the co-presence of CLO impaired the Fludarabine-induced apoptosis (5/5 CLL samples, Fig. 1E). As for CLO treatment, a trend for impaired fludarabine-induced cytotoxicity was observed in 5/5 and 4/5 CLL cases in presence of HEDP and ZOL, respectively (Supplementary Fig. S3). Similarly, in presence of CLO a trend for resistance to treatment of activated CLL B cells was observed for: (i) the pro-apoptotic effects on CLL B cells induced by Venetoclax (5/5 CLL samples, Fig. 1E); and (ii) the proliferative rate of CLL B cells treated with Ibrutinib (5/5 CLL samples, Fig. 1E). Hence, these in vitro data suggest that BPs might counterproductively reduce, in the activated fraction of CLL B cells, the effectiveness of a broad range of drugs routinely used for CLL therapy such as Fludarabine, Ibrutinib and Venetoclax.

In addition, we tested pamidronate (PAM) and risedronate (RIS) at sub-cytotoxic concentrations (10 μM and 1 μM respectively) as well [17, 33]. Evaluating four of the ‘responding’ CLL samples we found that both ‘pro-survival’ (4/4 CLL samples for PAM and 3/4 for RIS) and ‘pro-proliferation’ (3/4 CLL samples for PAM and 3/4 for RIS) effects were induced by these drugs as well, thus supporting a mechanism of action on CLL B cells likely common to most bisphosphonates (Supplementary Fig. S4).

Of note, the CLL B cells responsiveness to BPs in terms of increased proliferation and resistance to apoptosis was directly linked in most of the samples (Fig. 1F). Also, samples responsive to one BP were usually responsive to the other two as well, as demonstrated by the observation that the high CLO-responders (samples displaying CLO-mediated cell proliferation > 50% of untreated control) also showed a pronounced response to HEDP or ZOL (Fig. 1F).

### Poorer clinical course is linked with BP-responding CLL B cells

**Fig. 2:**
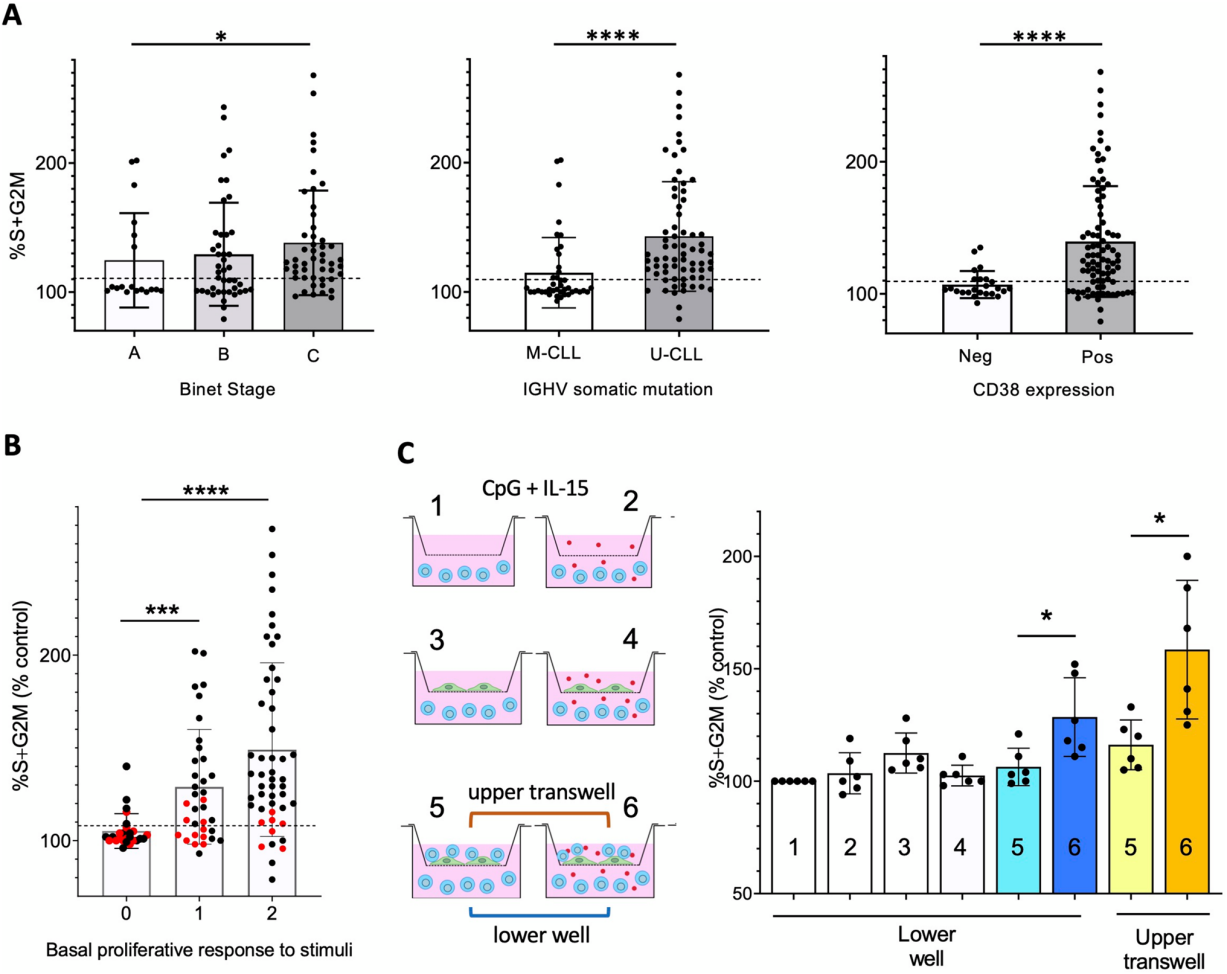
*Enhancement of CLL cell proliferation by BPs is prominent in aggressive CLLs, requires basal proliferation and presence of bystander cells, and is mediated by cell–cell contact and released soluble factors.* **A** Data on the proliferative response to CLO, HEDP and ZOL displayed in Fig. 1D were pooled together and clustered according to clinical and molecular features of the CLL, namely disease stage (Binet A, B or C), Ig mutational status (unmutated, U-CLL, < 2% mutations in *IGVH*) or mutated, M-CLL, ≥ 2% mutations in *IGVH* genes) and CD38^+^ cells (cut-off for positivity: ≥ 30% positive cells). Statistical significance of the difference is assessed by two-sided Mann–Whitney test. For the disease stages, significance line is related to Binet A versus Binet C comparison. **B** The data of BPs response in Fig. 1D were clustered accordingly to the level of basal proliferative response to the microenvironment stimuli (either CD40L + IL-4 or CpG + IL-15). The latter is expressed as % of cells in S + G2M at 4 days after stimulation. Level = 0 if %S + G2M ≤ 6; level = 1 if 6 < %S + G2M ≤ 15; level = 2 if %S + G2M > 15. Enhanced proliferation by BPs is expressed as % of S + G2M with respect to untreated control. CLL samples depicted by dots in red color are the samples devoid of NLC and stimulated with CpG + IL-15. Statistical significance of the difference is assessed by two-sided Mann–Whitney test. **C** Scheme and results of the transwell assay implemented to evaluate whether bystander cells prime CLL ability to respond to BPs by soluble factors or by direct cell/cell interactions. CLO-responding CLL samples devoid of NLC (pts # 5, 8, 9, 12, 14, 21), were seeded on the bottom of a well (azure circles) and were stimulated with CpG + IL-15 to elicit the cell-cycle entry required for the CLO-effect to occur (setting 1). CLO (30 μM, red dots) was added to the culture (setting 2). Fibroblasts (green shapes) alone (settings 3 and 4) or fibroblasts + CLL cells (settings 5 and 6) were seeded on the upper transwell-insert equipped with membranes containing 1 micron diameter pores. CLO (30 μM) was added in settings 4 and 6. To reproduce the standard CD40L + IL-4 activation setting, fibroblast:CLL were seeded at 1:100 ratio. In all conditions, % of S + G2M cells was measured by flow cytometry and expressed as % increase over control (setting 1). Wilcoxon matched-pairs test was used to evaluate the significance of the difference between samples without and with CLO in setting 6, either the lower well, or in the upper transwell

Based on the above data, specific concentration ranges of BPs seem to exert protective and proliferation-promoting effects in a relevant percentage of stimulated CLL samples. Thus we questioned whether these 'BP-responder' CLLs share clinical and biological characteristics. To this end, we clustered the CLL BP-responses of our cohort based on clinical stage (Binet stage) and prognostic markers (IGVH mutational status and CD38 levels). The CLL B cell response to BP was higher in samples of patients with unfavorable Binet disease stage, unmutated IGHV, and higher CD38 membrane levels (Fig. 2A). Thus, the CLL cases derived from patients with a more aggressive CLL disease were more sensitive to the survival-protective and proliferation-promoting activity of BPs (Fig. 2A).

Most interestingly, we found that the BP effect was higher in samples that more effectively entered the cell cycle in response to the in vitro activation stimuli (Fig. 2B). Conversely, BPs were not ‘effective’ in samples that did not respond to the stimuli and remained in a quiescent state (Fig. 2B).

### Accessory cells elicit the BP-response capacity of CLL

While further addressing the latter observation, we noticed that potentiation of proliferation by BPs was not effective in CpG + IL-15-stimulated cultures that lacked autologous monocyte-like Nurse-Like cells (NLCs) often recovered in CLL samples and remaining in culture attached to the bottom-well (albeit at very low density compared to the number of CLL B cells in culture). These NLC-devoid samples are highlighted in Fig. 2B in red and are mostly in the non-responder region (i.e., ≤ 110% of untreated stimulated control). Conversely, most of the same CLL samples responded to BPs when stimulated with CD40L + IL-4 (namely in the presence of fibroblasts). Therefore, it appears that two conditions were necessary (not sufficient) to elicit the pro-activation phenomenon: a positive basal activation/proliferation response of the CLL B cell to the ‘microenvironment’ stimuli (either CD40L + IL-4 or CpG + IL-15) and the presence of accessory ‘microenvironment’ bystander cells in the culture (either murine fibroblasts or autologous NLCs).

Experiments with transwells were performed to understand if the observed accessory cells prime CLL ability to respond to BPs by soluble factors released in the medium or by direct cell/cell interactions (Fig. 2C).

CLL B cells from CLO-responding samples devoid of NLCs (thereby responding to CLO only in the CD40L + IL-4 activation setting, not in the CpG + IL-15 setting) were seeded on the bottom of a well and stimulated with CpG + IL-15 to elicit cell-cycle entry, required for CLO-responsiveness. CLL B cells from the same sample were also seeded on an upper transwell insert that was permeable to drugs but not to cells, in the presence of fibroblasts. The addition of CLO to the system increased cell activation/proliferation of CLLs in the transwell. Importantly, although at a lower degree, CLO also increased proliferation of the CLLs on the well bottom, which were not in contact with bystander cells (Fig. 2C). CLO effects on these bottom-well CLLs were not observed when either fibroblasts or CLL cells were present alone—not in combination/contact—in the transwell (Fig. 2C).

Altogether, these data suggest that the BP effects may require cell–cell contact, but can also be caused by soluble factors released in the medium by CLL cells when they interact with the bystander cells or by the bystander cells themselves.

Due to the significant role of accessory cells in the CLL prosurvival/proliferation effects induced by BP, we investigated whether BPs would also directly target fibroblasts or NSCs, potentially enhancing their proliferation akin to what is observed in the CLL B cell counterpart. We conducted a comparison between control and CLO, HEDP, and ZOL-treated fibroblasts to assess alterations in their cell cycle. No significant differences were observed, indicating that BPs do not affect fibroblast proliferation (Supplementary Fig. S5).

Similarly, NSCs, which do not exhibit proliferation in culture but nonetheless support the effects of BPs on CLL B cells, were assessed for reduced spontaneous apoptosis or cell cycle entry in CLO, HEDP, and ZOL-treated cultures. As with fibroblasts, no disparities were observed between control and BPs-treated cells, neither in apoptosis rates nor in cell cycle induction (Supplementary Fig. S5).

Finally, we investigated whether the presence of autologous T or NK cells, present in the CLL sample, would exert an influence on the CLL BP-induced survival and proliferation. To address this, we conducted a comparison between patient-matched unpurified (Bulk) and purified CLL B cells samples treated with CLO. The presence or absence of NK/T cells did not alter the BP-induced effects on CLL, neither in terms of cell cycle distribution nor CFSE decay (Supplementary Fig. S6).

### BP-pro-survival/proliferative effects are independent of RANK/RANKL loop and purinergic responses

**Fig. 3:**
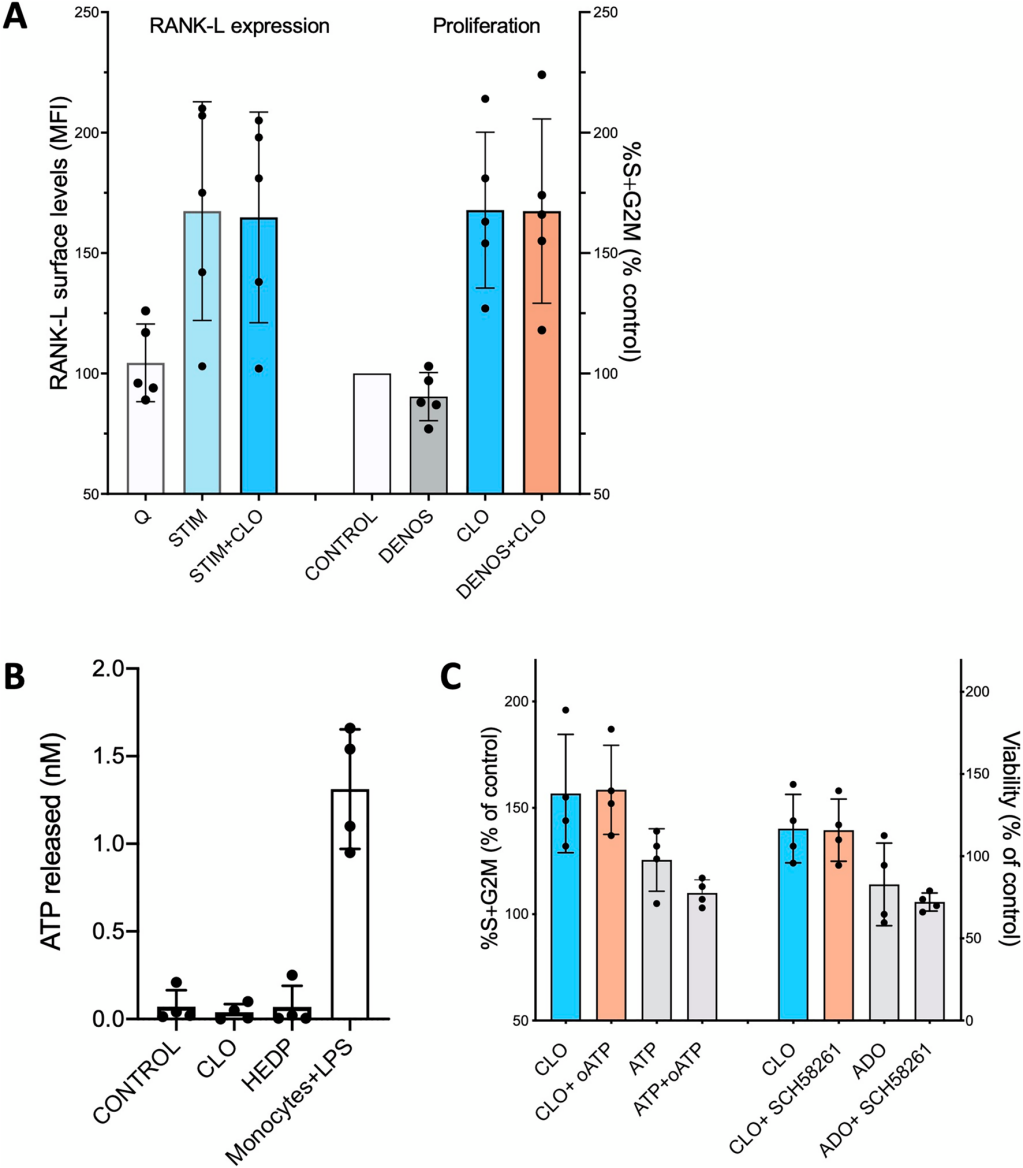
*BP enhancement of cell survival and proliferation does not utilize the RANK/RANKL loop and the purinergic pathway. A* Left: flow cytometric evaluation of RANKL expression of n = 5 stimulated CLO-responding CLL samples (pts # 5, 8, 11, 12, 14), either quiescent (‘Q’) or stimulated (‘Stim’) in the absence or presence of CLO. Right: cell proliferation in stimulated CLL samples treated with CLO in the absence or presence of the anti-RANKL antibody Denosumab that impairs binding of RANK to RANKL and thus the activation of the RANKL pathway. Changes in proliferation were defined as % of untreated control (i.e. 100% = control, < 100% less proliferation than control, > 100% more proliferation than control). **B** Concentration of ATP released in the culture medium evaluated after 4 days of CLO (30 μM) and HEDP (3 mM) treatment of n = 4 stimulated CLL samples (pts # 11, 12, 14, 21). Monocytes samples (n = 4) from healthy donors, which were activated with LPS, were used as positive controls. **C** Left: CLO-responsive CLL samples were stimulated with CD40L + IL-4 and treated with CLO, in the absence or presence of oATP, which inhibits the activation of the ATP-receptor P2X_7_R. Changes in proliferation were defined as % of untreated control. Positive controls were obtained by treating the samples with ATP in the absence or presence of oATP. Right: CLO-responsive CLL samples (pts # 11, 12, 14, 21) were stimulated with CD40L + IL-4 and treated with CLO, in the absence or presence of the ADO inhibitor SCH58261. Viability is expressed as % of its respective untreatied control. Positive controls were obtained by treating the samples with 2-chloroadenosine in the absence or presence of the inhibitor. Histogram plots with mean ± SD are shown

ZOL was observed to increase gene expression of RANKL in human osteoblasts [34]. RANKL is expressed on the surface of CLL cells as well [35] and activates pathways that promote CLL B cell survival and proliferation when bound by RANK, which is also expressed by CLL cells [35, 36]. Hence, we wondered if the BP stimulatory effect on CLL cells could be mediated by increase of the autocrine/paracrine RANK/RANKL loop activity. However, we found that surface RANKL levels in CD40L + IL-4-stimulated CLL B cells were unaffected by CLO treatment (Fig. 3A). Also, the BP-mediated increase of proliferation was unperturbed by the presence of Denosumab, an antibody that blocks the interaction between RANKL and RANK (Fig. 3A). These observations suggest that the BP-mediated pro-activation phenomenon is likely independent of the RANK/RANKL loop.

BPs were observed to prevent apoptosis of osteoblasts and induce their proliferation by triggering the release of non-lytic ATP that promotes mitogenic signals by binding to purinergic receptors [33, 37]. On lymphoid cells, low-levels of extracellular ATP induce their growth by binding and activating the ATP–gated plasma membrane channel P2X_7_ receptor (P2X_7_R) [38]. Interestingly, CLL cells, particularly the aggressive cases, display high membrane levels of P2X_7_R whose expression is further increased when CLL cells are stimulated in vitro [39]. Hence, we investigated whether a similar mechanism was involved in the BP-survival and -proliferative actions. Initially, we measured ATP levels in the medium of CLL cultures treated with CLO and HEDP and found a lack of extracellular ATP comparable to that measured in the medium of untreated CLLs (Fig. 3B). Then, we tested whether inhibition of the binding of extracellular ATP to P2X_7_R, obtained with a competitive selective and irreversible inhibitor (oxidized ATP, oATP), could interfere with the CLO-potentiation effects. The CLO-mediated increase of CLL activation was unbiased by the presence of the inhibitor in the cultures (Fig. 3C).

It has to be considered that CLL cells express surface ectonucleotidases CD39 and CD73, which are able to convert extracellular ATP (and ADP) into AMP, and AMP into adenosine (ADO), respectively [40]. CD39 is constitutively expressed, while CD73 levels are variable and increase when cells are activated [40]. Thus, we evaluated whether extracellular ATP levels were decreased by conversion to ADO, known to protect CLL B cells from spontaneous apoptosis by binding and activating the ADO receptor P1A2A [40]. The latter is highly expressed by CLL cells and could play a role in the CLO-mediated protective activity. Accordingly, we studied the ‘BP-phenomenon’ in CLL samples with the P1A2A receptor impaired by a specific antagonist (SCH58261). However, we did not observe any impairment of CLO-induced rise of viability (Fig. 3C). This may suggest that the mechanism of CLO-potentiation of CLL B cell survival operates in absence of extracellular ATP enzymatically converted to adenosine. Of note, the receptors P2X_7_R and P1A2A were fully functional, as both ATP- and ADO- mediated potentiation of proliferation and survival, respectively, were sensitive to the inhibitors of the two respective receptors (Fig. 3C).

Altogether, the BP-phenomenon occurring in CLL B cells does not require activation of purinergic responses, neither by released ATP nor by its converted nucleotide counterpart.

## Discussion

B cell chronic lymphocytic leukemia presents on average at 65–70 years of age at the time of diagnosis, and is uncommon in younger individuals (≤ 40 years of age) [2, 3]. Thus, patients with CLL often display comorbidities whose presence associates with poorer outcomes and requires co-treatment of the various conditions [4, 5]. Osteoporosis, among the possible co-existing conditions, may also be induced or aggravated by the leukemic cells [6, 7]. In this context, bisphosphonates represent a broad class of drugs commonly prescribed for the treatment of osteoporosis [10]. Despite comprehensive studies about the overall use of BPs in CLL are currently missing, a recent report showed that in a US Medicare cohort of 10,834 CLL patients at least 1450 (12.5%) received BP treatment associated with osteoporosis comorbidity [8] and a recent CLL database reports 31% CLL patients that received BP treatment for osteoporosis [9].

Some BPs (e.g., CLO, HEDP, ZOL, PAM, etc.), were shown, in various preclinical studies, to possess antitumor activity [12, 13, 16, 41–43], including in vitro cytotoxicity effects of ZOL and PAM on CLL B cells [17].

Herein, investigating more deeply the effects of BPs on CLL B cells, we observed opposite cellular consequences upon treatment with different concentrations. Specifically, either cytotoxicity or survival/proliferative effects were observed on activated CLL B cells, respectively at higher or lower BP concentrations. BPs mediated cytotoxicity at drug concentrations above 100 μM for CLO, 10 mM for HEDP, and 30 μM for ZOL, in agreement with a previous study on ex vivo CLL cells treated with 50 to 100 μM of ZOL and PAM [17]. However, at lower doses, specifically in a limited range around 30 μM for CLO, 3 mM for HEDP, and 10 μM for ZOL, CLL B cells activated by microenvironmental stimuli responded with increased survival and enhanced proliferation.

These same BP concentrations increased chemoresistance; in particular, it decreased Fludarabine-induced cytotoxicity. A trend towards reduced efficacy during treatment with Venetoclax and Ibrutinib was also noted, and increased survival and cell proliferation mediated by BPs were directly associated with markers of aggressive clinical course.

This kind of response of CLL B cells to the BPs was associated with the presence of bystander cells (i.e., autologous Nurse-like cells or xenogenic fibroblasts). This is in line with the knowledge of CLL being a disease that requires support and cross-talk, by direct intercellular contacts or release of soluble elements, within the lymphoid microenvironment (e.g., lymph nodes and bone marrow). However, the observed effects were not due to a direct effect on cell growth of the stromal bystander cells present in the culture. To be specific, fibroblasts and NSCs treated with CLO, HEDP, and ZOL showed no significant alterations in cell cycle nor survival. Additionally, the depletion of autologous T or NK cells from BP-responding CLL samples did not alter the BP-induced effects on the CLL B cells. These data indicate that BPs do not act through direct modifications of the microenvironmental niche required to activate the leukemic cells. Rather, BPs induce the enhanced prosurvival/proliferation phenomenon by specifically targeting the activated CLL B cells previously primed by the crosstalk with fibroblasts or NSCs. These results agree with an in vivo study on B cell activation by CLO [44]. Administration of clinically relevant doses of the bisphosphonate in mice was able to increase activation of B cells in response to antigenic stimuli. Interestingly, this activity was independent of T cells, inflammatory monocytes, neutrophils, or dendritic cells, indicating that BPs directly targeted B cells [44].

Attempting to dissect the ongoing mechanism utilized by the CLL B cells to crosstalk with the microenvironment and acquire the BP-responsiveness characteristics, we found that enhanced survival and proliferation of CLL B cells in presence of BP, is likely mostly dependent on cell-to-cell contact but could also be triggered by soluble factors released in the medium by CLL cells when they interact with the stromal bystander cells or by the bystander cells themselves. In this context, we focused on targeted analysis based on the state-of-art knowledge of BPs mechanism of action in osteoblasts, and evaluating the role of RANK/RANKL and purinergic pathways, known for their importance both in CLL progression and osteoporosis [33–40], we demonstrated that the BPs effects observed do not operate through these conventional pathways. Hence, the specific elements of the lymphoid microenvironment that may participate—by cellular contact, autocrine, and/or paracrine mechanisms—in mediating the in vitro observed pro-activation and pro-proliferation effects of BPs on CLL cells remain unsettled.

The BP concentrations described herein to promote increased CLL cell proliferation might have a clinical relevance. The available pharmacokinetic studies carried out on patients treated with BP for osteopenia/osteoporosis report that the bloodstream exhibits a brief peak BP concentration followed by rapid and consistent clearance [18, 45–48]. While concentration ranges vary among different BPs, most share these pharmacokinetic features [18, 49–51]. For example, ZOL reaches a peak blood concentration of approximately 1 mM, which rapidly declines to 3–10 μM [46, 52, 53]. CLO serum levels range from 2 to 10 μM [48]. Risedronate levels never exceed 3–10 μM [54], while pamidronate levels remain below the μM range [51, 55]. In contrast, BPs tend to accumulate in calcified tissues at high concentrations, reaching levels of up to 100–1000 μM depending on the specific BP and method of administration [18, 49–51]. In summary, non-calcified tissues have significantly lower BP concentrations compared to bone tissues [18, 47]. This suggests that most of the time, BP levels in the blood remain in a steady-state 'low' range. It is also reasonable to hypothesize that in other non-calcified tissue where CLL B cells reside and interact with their microenvironment, such as lymph nodes [20, 56], BP concentrations are like those in the blood.

Thus, accordingly with the previous studies that used high BP concentrations, the cytotoxic effects on neoplastic cells are likely achievable for treatment of bone cancers and/or bone metastasis (e.g., multiple myeloma, breast cancer) [57, 58] and not for malignancies that preferentially reside in non-calcified tissues.

While unable to identify the molecular mechanism of action of BPs on the CLL B cells, this study shows potentially important pro-leukemia effects in the context of BP concentrations that are likely achievable in lymph nodes where CLL cells reside and receive microenvironment stimuli, and identify the role of the microenvironment and stimulatory pathways in the survival/proliferation of CLL B cells exposed to BPs. The portray of this phenomenon could be important to prompt further studies on this issue. Given that both disease and treatment courses have a very heterogeneous outcome among CLL patients, these future studies should include a broad cohort of CLL patients for multicenter clinical trials and/or retrospective metanalysis.

### Supplementary Information

Below is the link to the electronic supplementary material.

Supplementary file1 (PDF 2313 kb)

## Data Availability

The datasets generated during and/or analysed during the current study are available from the corresponding author on reasonable request.
