# Supplementary material for: Unexpected chronic lymphocytic leukemia B cell activation by bisphosphonates
Source: Cancer Immunol Immunother. 2024 Jan 27;73(2):27. doi: 10.1007/s00262-023-03588-z (PMC10821833; doi:10.1007/s00262-023-03588-z)
Supplement: Supplementary file 1 — Supplementary file1 (PDF 2313 kb) [file 262_2023_3588_MOESM1_ESM.pdf]

Unexpected Chronic Lymphocytic Leukemia B Cell Activation by Bisphosphonates.

Andrea N Mazzarello[1], Elena Gugiatti[1], Vanessa Cossu[1], Nadia Bertola[2], Davide Bagnara[1], Sonia Carta[3], Silvia Ravera[1], Chiara Salvetti[4], Adalberto Ibatici[5], Fabio Ghiotto[1,2], Monica Colombo[2], Giovanna Cutrona[2], Cecilia Marini[3,6], Gianmario Sambuceti[3,7], Franco Fais[1,2,*], Silvia Bruno[1,*,#]

1. Department of Experimental Medicine (DIMES), University of Genoa, Genoa, Italy
2. Molecular Pathology Unit, IRCCS Ospedale Policlinico San Martino, Genoa, Italy
3. Nuclear Medicine Unit, IRCCS Ospedale Policlinico San Martino, Genova, Italy
4. Clinic of Hematology, IRCCS Ospedale Policlinico San Martino, Genoa, Italy
5. Division of Hematology and Bone Marrow Transplant, IRCCS Ospedale Policlinico San Martino, Genoa, Italy
6. Institute of Molecular Bioimaging and Physiology (IBFM), National Research Council (CNR), Milan, Italy
7. Department of Health Sciences (DISSAL), University of Genoa, Genova, Italy

* Franco Fais and Silvia Bruno contributed equally to this work.

# corresponding author

Silvia Bruno Ph.D.
University of Genoa
Department of Experimental Medicine
Via De Toni 14
16132 Genova, Italy
Office: +39 010 335 7876
Email: silvia.bruno@unige.it

Key words: Chronic Lymphocytic Leukemia (CLL), Bisphosphonates, CLL cell activation, CLL cell treatment.

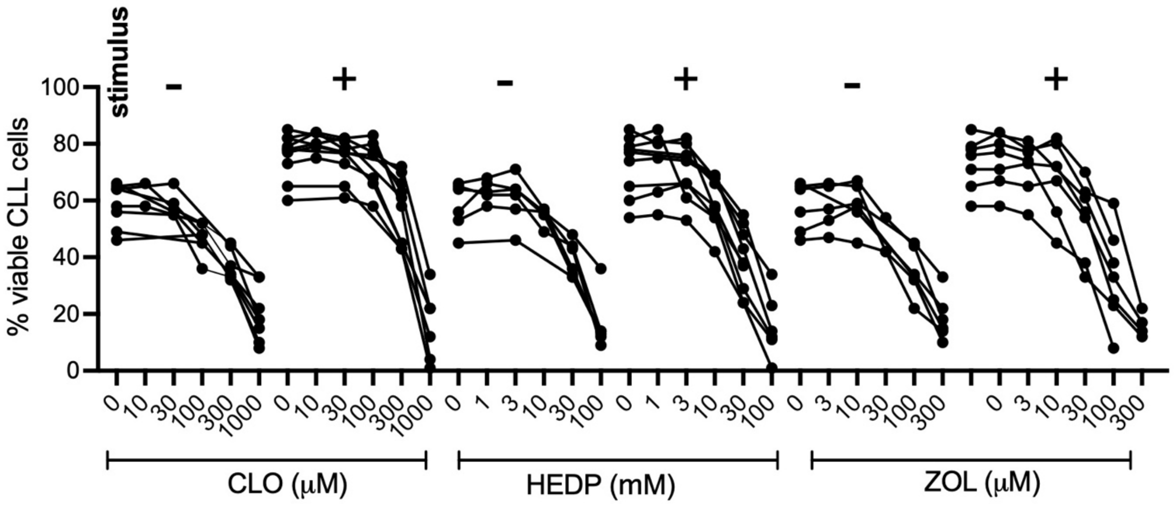

Figure S1. *Dose-dependent inhibitory effects of BPs on CLL viability in absence or presence of microenvironment-mimicking stimuli.* Cellular viability expressed as % of negative cells as assessed by propidium iodide (PI) exclusion assays after 3 days of *in vitro* culture of CLL B samples cultured in absence (-) or in presence (+) of microenvironmental stimuli with BPs at the reported concentrations. Clodronate (CLO; Etidronate (HEDP); Zoledronate (ZOL).

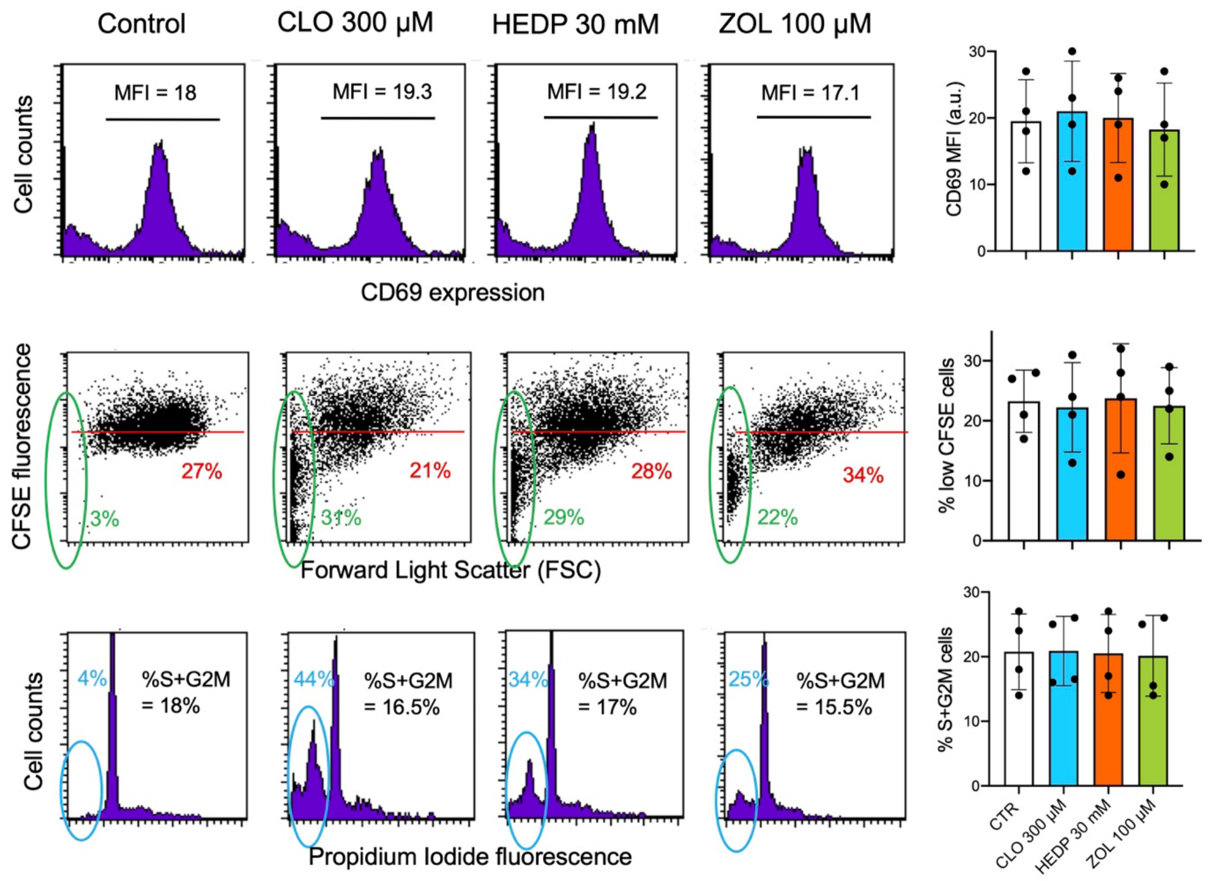

Figure S2. *High BP concentrations affect CLL cell survival, not CLL cell proliferation.* Effect of CLO (300 μM), HEDP (30 mM) and ZOL (100 μM) on leukemic cell proliferation of one CLL sample selected among those displaying 'pro-survival' response to BPs (pt. # 12). The sample was stimulated with CD40L+IL-4 (Control) and treated with BPs for 3 days. On the right-side, summary of data on 4 BP-responding CLL samples (pt. # 8, 11, 12, 14).

Upper: flow cytometric histograms of CD69 expression of CLL cells gated on the 'live' gate (PI-negative fluorescence).

Middle: CFSE fluorescence vs Forward Scatter dot plots, reporting the % of cells with diluted CFSE fluoresce in red (i.e., below the threshold based on unstimulated Quiescent cells). Dead cells/debris with low FSC values (green gate). The % of cells with diluted CFSE is normalized to the viable cell population (thus excluding dead cells/debris).

Lower: DNA content histograms showing the sub-G1 peak on the left representing the apoptotic cells/bodies (% indicated in blue color) and the cell cycle distribution whose reported %S+G2M (representative of cell proliferation) is normalized to the 'viable' cell population (thus excluding the sub-G1 peak).

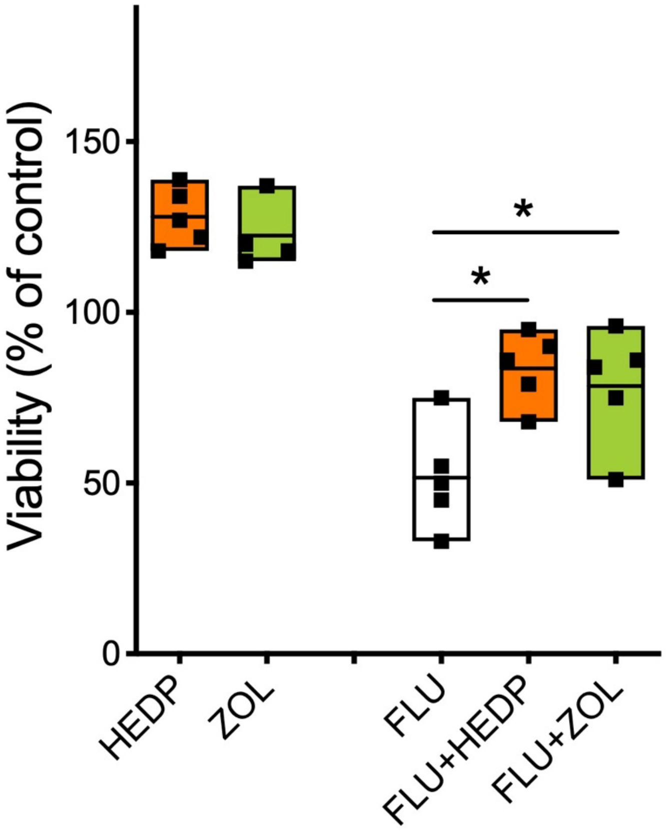

Figure S3. *BPs inhibit fludarabine-induced cytotoxicity.* **A)** Cellular viability of n=5 CLL samples selected among those displaying 'pro-survival' response to BPs. Samples were stimulated and cultured with Fludarabine (2.5 μM) in the absence or presence of (3 mM) HEDP (3 mM) or ZOL (10 μM), for 4 days. Viability of treated samples is expressed as % of untreated controls. Groups are shown as bars with data mean and range (min to max). Wilcoxon matched-pairs test was used to evaluate the significance of the difference between Fludarabine-treated group without BPs and with BPs.

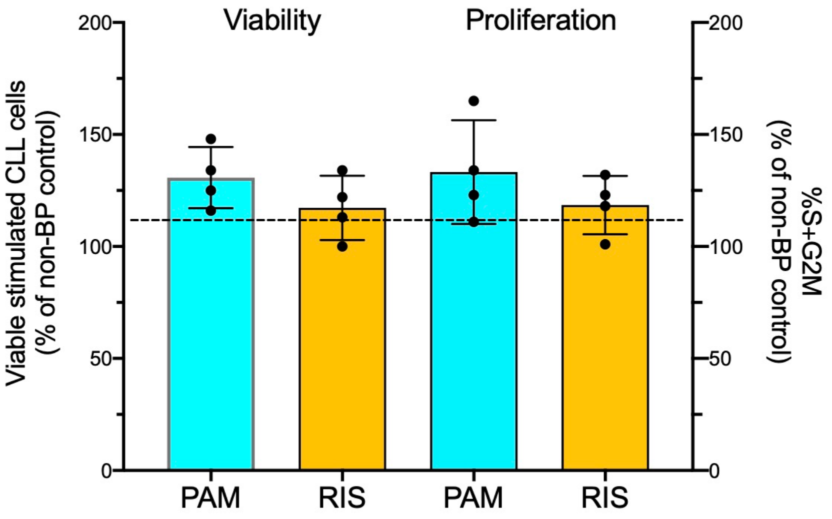

Figure S4. *Survival and proliferative effects of Pamidronate and Risedronate on activated CLL samples.* CLL B cells were activated by microenvironmental-mimicking stimuli in absence or presence of Pamidronate (PAM, 10 μM) and Risedronate (RIS, 1 μM). Left: Viability expressed as % viable cells in BP-treated samples divided by % viable cells in control untreated samples (i.e., % of control). Right: proliferation expressed as % S+G2M cells in BP-treated samples divided by % S+G2M cells in control untreated samples (i.e., % of control). Histogram plots with mean ± SD are shown. Dotted line indicates the value of 110%, defined as the operational threshold for the determination of positive 'BP-mediated effect'.

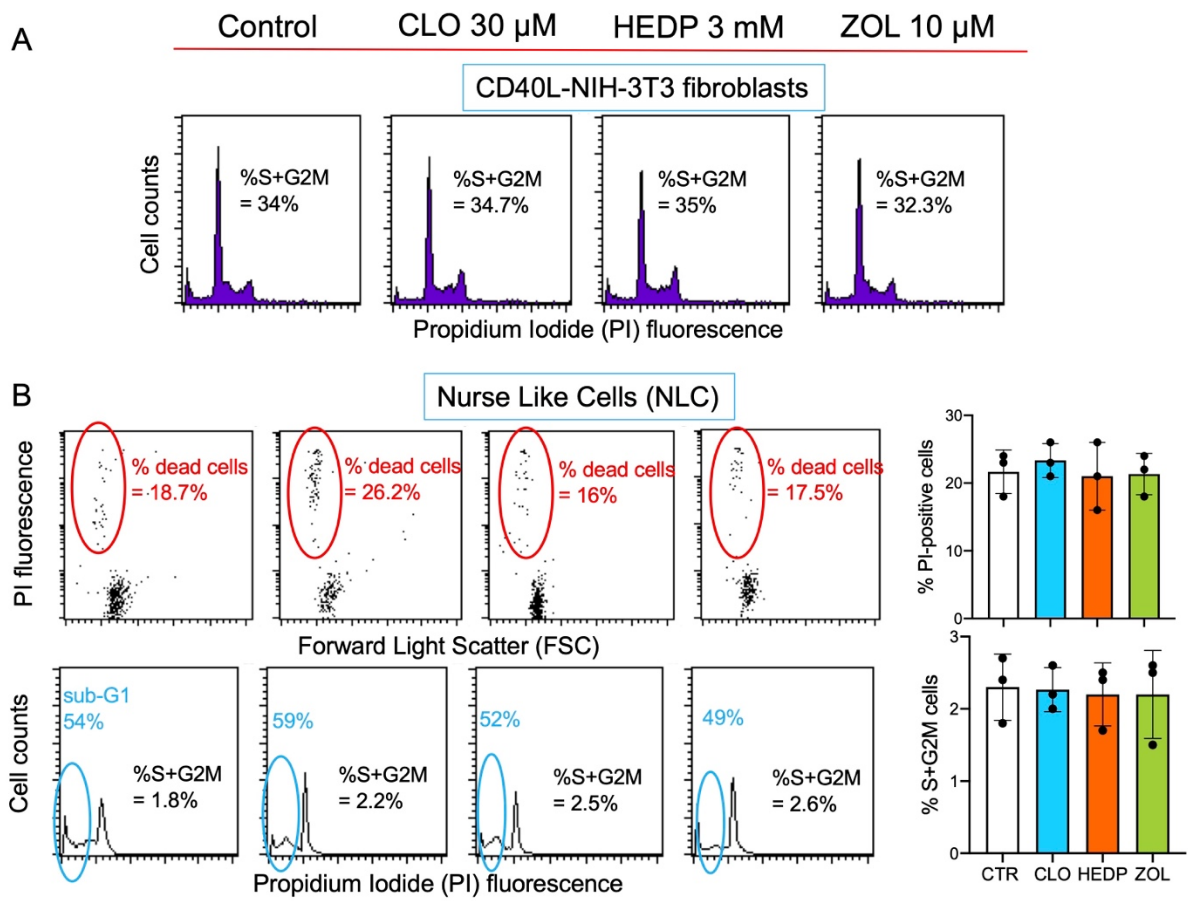

Figure S5. *BPs do not affect survival and proliferative status of murine fibroblasts nor of CLL sample-derived monocytic cells (NLC).* A) Flow cytometric DNA histograms of CD40L-expressing NIH-3T3 cells cultured with BPs at the same concentrations observed to trigger the pro-activation phenomenon in CLL cells for 5 days. No changes in cell proliferation are detected, as indicated by the % of cells in the S+G2M cell cycle phase. B) Cell viability of NLCs is evaluated by flow cytometric PI-exclusion assay (upper) and cell cycle phase distribution by flow cytometric DNA fluorescence histograms (lower). To obtain NLC cells, CLL samples (N=3) containing a significant amount of NLC (around 2% of total cells) were seeded in culture. CLL#6 is shown as representative result. After 24 hours all cells in suspension were removed and the sparsely attached cells, NLC of the monocytic lineage were treated with BPs. No proliferation induction nor apoptosis protection were observed with neither BP.

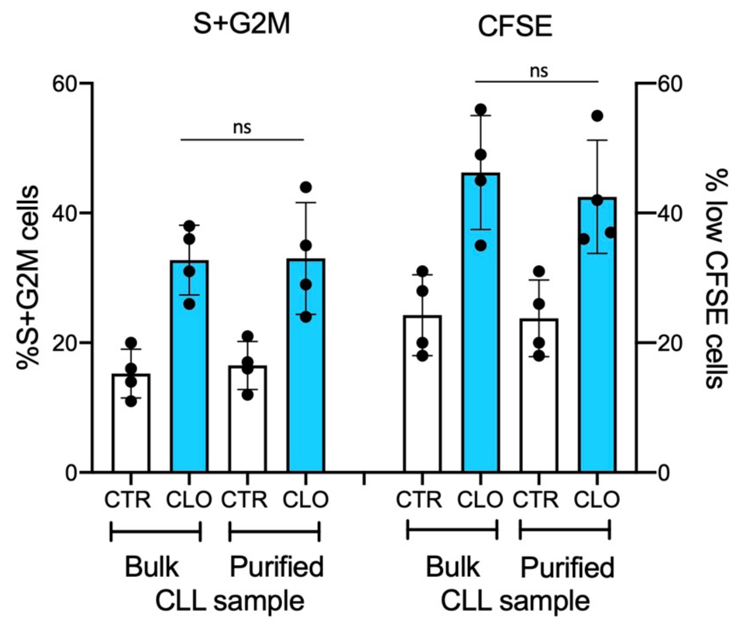

Figure S6. *Deprivation of T and NK cells from CLL samples did not affect the BP-mediated increase of leukemic cell proliferation.* Four CLO-responding CLL samples (patients # 5, 11, 12, 14) were subjected, by magnetic immunobeads, to depletion of the T and NK cell pool (around 10%) still present in the sample ('purified'). Their proliferative response to CLO was then compared with that of the respective original CLL sample ('bulk'), by flow cytometric analysis of cell cycle distribution (left) and CFSE dilution (right). No significant differences were observed.

CLL #	Binet Stage	IGHV Mut Status	IGHV gene usage	CD38	TP53	NOTCH1	SF3B1	FISH
1	C	M	VH1-2*02	POS	NA	NA	NA	del 17p13 (5%)
2	A	M	VH3-23*01	NEG	WT	NA	NA	del13q14
3	A	U	VH4-39*01	NEG	EX7 c.704a>G,P, Asn235Ser	NA	WT	trisomy 12
4	B	M	VH3-48*02	NEG	NA	NA	NA	del 13q14
5	C	U	VH1-69*D01	POS	WT	WT	WT	del 13q14, trisomy 12
6	C	U	VH5-51*03	POS	WT	WT	WT	NA
7	B	M	VH3-21*01	POS	WT	WT	WT	del13q14
8	A	M	VH3-48*02	POS	WT	NA	NA	del 13q14
9	C	M	VH1-18*01	POS	WT	NA	NA	del 13q14, del 17p13
10	B	M	VH4-31*03	POS	WT	WT	WT	NA
11	B	U	VH1-69*01	POS	WT	WT	WT	NA
12	C	M	VH3-30*03	POS	WT	WT	WT	del 11q22, del 13q14, del 17p13
13	B	U	VH3-11*01	NEG	WT	WT	WT	del 13q14
14	B	U	VH3-74*01	POS	WT	WT	MUT(pGly 742Asp)	del 17p13
15	A	U	VH4-34*01	POS	WT	NA	NA	trisomy 12, del 17p13
16	B	U	VH1-18*04	NEG	WT	NA	NA	NEG
17	A	M	vh3-23*01	NEG	WT	WT	WT	del 13q14
18	B	M	VH3-7*01	POS	WT	NA	NA	trisomy 12
19	B	M	VH3-7*01	POS	WT	NA	NA	trisomy 12 (45%), del 13q14 (6%), del 17p13 (4%)
20	C	U	VH3-23*04	NEG	MUT ex4 silent	WT	MUT (p.His662 Gln)	del 13q14
21	C	U	VH4-74*01	POS	mut ex10	WT	WT	del 11q22, del 13q14

Table S1. *Clinical characteristics of the patients with CLL analyzed in this study.* For each patient, Binet stage; IGHV mutation status (≤2% mutations, U = IGHV-unmutated; >2% mutations, M = IGHV-mutated); IGHV gene; CD38 (cut-off > 30% = POS); TP53 mutant status; NOTCH1 mutant status; SF3B1 mutant status; genetic abnormalities defined by fluorescent in situ hybridization (FISH, Positive if observed > 10% cells). WT = Wild Type; NA = Not Available; NEG = Negative; POS = Positive.
